# Comparative effects of CFTR modulators on phagocytic, metabolic and inflammatory profiles of CF and nonCF macrophages

**DOI:** 10.1038/s41598-023-38300-9

**Published:** 2023-07-25

**Authors:** Daniel S. Aridgides, Diane L. Mellinger, Lorraine L. Gwilt, Thomas H. Hampton, Dallas L. Mould, Deborah A. Hogan, Alix Ashare

**Affiliations:** 1 Section of Pulmonary and Critical Care Medicine, Dartmouth-Hitchcock Medical Center, Lebanon, NH USA; 2 Department of Microbiology and Immunology, Dartmouth College, Geisel School of Medicine, Hanover, NH USA

**Keywords:** Immunology, Antimicrobial responses, Infection

## Abstract

Macrophage dysfunction has been well-described in Cystic Fibrosis (CF) and may contribute to bacterial persistence in the lung. Whether CF macrophage dysfunction is related directly to Cystic Fibrosis Transmembrane Conductance Regulator (CFTR) in macrophages or an indirect consequence of chronic inflammation and mucostasis is a subject of ongoing debate. CFTR modulators that restore CFTR function in epithelial cells improve global CF monocyte inflammatory responses but their direct effects on macrophages are less well understood. To address this knowledge gap, we measured phagocytosis, metabolism, and cytokine expression in response to a classical CF pathogen, Pseudomonas aeruginosa in monocyte-derived macrophages (MDM) isolated from CF F508del homozygous subjects and nonCF controls. Unexpectedly, we found that CFTR modulators enhanced phagocytosis in both CF and nonCF cohorts. CFTR triple modulators also inhibited MDM mitochondrial function, consistent with MDM activation. In contrast to studies in humans where CFTR modulators decreased serum inflammatory cytokine levels, modulators did not alter cytokine secretion in our system. Our studies therefore suggest modulator induced metabolic effects may promote bacterial clearance in both CF and nonCF monocyte-derived macrophages.

## Introduction

Cystic Fibrosis (CF) is a multisystem genetic disorder in which respiratory disease plays a prominent role in morbidity and mortality^1^. People with CF (pwCF) are plagued by chronic lung infections, often beginning with stereotypical pathogens *Staphylococcus aureus* and *Haemophilus influenzae*, followed frequently by a transition to colonization with *Pseudomonas aeruginosa* (Pa), other bacteria and fungi as pwCF age and lung disease progresses^2^. This process is classically understood to arise from a cascade beginning with a lack of CFTR-mediated chloride secretion leading to resultant mucus hyper-viscosity, failure of the mucociliary escalator, and biofilm formation all culminating in a lack of sterilizing immunity within the lung^1^.

This model has been challenged somewhat by the real-world experience of pwCF who are on highly effective CFTR modulator therapy. For those with at least one copy of the most common CFTR mutation, a deletion in phenylalanine at position 508 (F508del), modulators can restore CFTR function to levels approaching those in people without CFTR mutations^3,4^. While modulators can cause a robust improvement in lung function, lung clearance, and decrease in sputum production and bacterial quantity, bacterial colonization has unfortunately remained in most subjects, and has the potential to drive further lung disease progression^5–7^. This has highlighted the need to understand whether there may be intrinsic immune defects in CF^8–12^. Macrophages serve as the gatekeepers of the immune system in the lung and investigating their response to CFTR modulators is therefore critical to understanding CF lung immunity more broadly^13^.

Prior investigations on serum monocytes from pwCF have looked at their profiles pre- and post-initiation of highly effective modulator therapy, both in those with gating mutations who started on ivacaftor and in those with F508del mutations starting on elexacaftor/tezacaftor/ivacaftor (ETI) triple combination therapy^6,14–17^. These studies have shown decreases in inflammatory cytokine production by CF monocytes however they cannot differentiate between direct effects of modulators on monocytes versus indirect effects secondary to improved mucociliary function and decreased bacterial burden. The earlier generation CFTR corrector lumacaftor was shown to improve CF macrophage phagocytosis and killing, whereas the CFTR potentiator ivacaftor decreased inflammatory cytokine secretion^18^. A more recent publication has shown effects of the newest generation triple CFTR modulators in vitro on isolated Monocyte Derived Macrophages (MDM)^19^. They found that while ETI increased bacterial clearance by CF MDM, it did not impact cytokine secretion in response to *Burkholderia cepacia* complex infection. We investigated the effects of ETI versus the older generation combination tezacaftor/ivacaftor (TI) on direct Pa uptake by CF and nonCF MDM, as well as the effects of modulators on MDM metabolism and cytokine production. Our results support a model whereby ETI activates macrophages and enhances bacterial clearance, rather than shifting them toward a less inflammatory phenotype as seen with prior modulator combinations.

Some of these results have previously been presented in the form of abstracts^20–22^.

## Materials and methods

### Human subjects

The Institutional Review Board at Dartmouth Health approved this study (IRB protocol #22781) and all methods were performed in compliance with relevant guidelines and regulations. Subjects were recruited from our CF clinic population if they were 18–65 years old, F508del homozygous, at clinical baseline without symptoms of exacerbation, and had normal hemoglobin levels. NonCF control subjects were recruited by IRB-approved flyers; smokers or those with respiratory disease or pulmonary medication use were excluded. Written informed consent was obtained from all subjects and recorded in a secure database. Phlebotomy was then performed (100 mL whole blood isolated) for monocyte isolation by our research nurse and taken directly to the laboratory for downstream processing. Clinical information on subjects is available in Table 1.

**Table 1:** Clinical characteristics of study participants.

	CF	nonCF
N	10	12
Percent Female	20%	75%
Age Mean (s.d.)	29 (4.5)	35 (11.9)
FEV1% predicted Mean (range)	82 (27–133)	
BMI Mean (s.d.)	25.9 (3.7)	
Pa positive	7/10	
Duration of Pa colonization in years Mean (s.d.)	3.7 (1.9)	
Other bacterial colonization		
Staphylococcus aureus	4/10	
Achromobacter xylosoxidans	1/10	
Moraxella catarrhalis	1/10	
CF-related diabetes	4/10	
Pancreatic insufficient	10/10	
Chronic azithromycin	4/10	
Inhaled aztreonam	2/10	
Inhaled tobramycin	0/10	
Nebulized DNAse	4/10	
Nebulized saline	3/10	
Annual outpatient exacerbation rate (past 3 years) Mean (s.d.)	0.27 (0.33)	
Annual hospitalized exacerbation rate (past 3 years) Mean (s.d.)	0.33 (0.33)	

MDM from a subset of subjects was used for each assay due to limitations on cell numbers from each isolation. All CF subjects were F508del homozygotes and on ETI at the time of blood draw.

### Generation of monocyte derived macrophages (MDM)

MDM were generated according to previously published protocols^23,24^. Briefly, monocytes were isolated from peripheral blood by Ficoll gradient separation followed by processing with a pan-monocyte isolation kit (Miltenyi Biotech) according to manufacturer’s protocols which produces a mixture of classical, non-classical and intermediate monocytes. They were quantified and plated directly onto tissue cultures plates as per intended assay. MDM were generated by treatment with 100 ng/mL M-CSF for 7 days in RPMI/10% FCS with gentamicin. Medium was exchanged every 2–3 days until cells were ready for use.

### MDM phagocytosis assay

MDM were generated at 2.5 × 10^5^/mL in 24-well plates. MDM were placed in RPMI/10% FBS with gentamicin without M-CSF, and CFTR modulators were added for 48 h. While there are no standard concentrations of CFTR modulators for in vitro use, we estimated effective concentrations based on previously published data and serum levels from people with CF. Elexacaftor was used at 6 µM, tezacaftor at 3 µM, and ivacaftor at 30 nM. Elexacaftor concentration was determined based upon published data on serum concentrations in CF subjects^25^, pharmacokinetic modeling data^26^, and package inserts of ETI following FDA approval^27^. Maximum elexacaftor concentration in these subjects was 9.2 µg/mL which corresponds to 15.4 µM. In vitro concentrations used on epithelial cells for the paper which first prompted the clinical approval of ETI were 2–3 µM^28^. Thus we are well within the range of concentration which was previously demonstrated efficacious as well as safe in vivo.

Similarly the maximum tezacaftor concentration in vivo was 7.7 µg/mL or 14.8 µM^27^. Independent analysis found a mean serum concentration of 5.1 µg/mL or 9.8 µM. Other in vitro studies have used between 5 and 18 µM^17,19^.

We chose a lower concentration of ivacaftor (30 nM) as prior work has demonstrated that it may have deleterious effects on phagocytosis rescue at higher concentrations^18^. Max concentration from the clinical package insert was 1.2 µg/mL (2.3 µM) and it is > 97% bound to plasma proteins in vivo^29^. Ivacaftor has been shown to be active on CFTR in vitro at concentrations as low as 10 nM^30^ and higher concentrations have been demonstrated to have a destabilizing effect on F508del CFTR^31^. Finally, preliminary experiments we conducted prior to the availability of elexacaftor demonstrated that 30 nM ivacaftor was sufficient to have an additive effect on enhancement of phagocytosis when used in combination with tezacaftor.

MDM were then washed with PBS and medium was replaced with identical medium without antibiotics. Fresh CFTR modulators were added with all media exchanges.

Pa strains PA14 or DH1137 (a non-mucoid CF clinical isolate) were grown overnight in LB at 37 °C in a shaking incubator with a loose cap tube angled at 45° to allow gas exchange. We chose both a standard laboratory strain which is broadly used in the literature, as well as a clinical isolate to ensure the effects were seen in a CF strain as well. Cultures were then sub-cultured 1:10 into LB for 60 min, centrifuged at 8000×*g* for 2 min, washed twice and then resuspended into antibiotic-free assay medium, and bacterial density was determined by spectrophotometry at 600 nm. OD600 of 1.0 was empirically determined to represent 10^9^ bacteria/mL media. Pa were added to MDM at a multiplicity of infection (MOI) of 10 bacteria per macrophage. Cell plates were incubated at 37 °C for 20 min, then cells were washed with PBS and MDM medium with 10 × gentamicin was added for 15 min. Cells were then washed twice with PBS at 4 °C followed by lysis with 250 µL 0.1% Triton X-100. 30 µL of lysate was then plated overnight onto LB agar plates for CFU determination. Colonies were counted the next day and bacteria per 10^6^ macrophages was calculated. Mean CFU for each subject and condition were then log-transformed. MOI was confirmed empirically from the stock bacterial solutions for each assay by plating onto LB agar plates.

### Seahorse extracellular flux assay

Monocytes were plated initially onto a Seahorse 96-well plate at 50,000 cells per well and differentiated into MDM directly in the wells for 7 days as above. At that point, differentiation medium was removed and CFTR modulators were added for 48 h. Pa conditioned medium was prepared in advance by culturing bacteria overnight in Seahorse mitostress assay medium, normalizing cultures to an OD600 of 0.500, centrifuging to pellet bacteria then passing supernatant through a 0.22 µm filter. This was defined as 100% conditioned medium. Aliquots were then frozen at − 20 °C until use.

On the day of assay, cell culture medium was exchanged for mitostress assay medium as per manufacturer’s instructions. A mitostress assay with acute injection was run on an Agilent Seahorse XFe96 platform, with 20 µL Pa conditioned medium in injection port A (final concentration 10% after injection), plain mitostress medium was included for control wells. Oligomycin, FCCP, and Rotenone/Antimycin A were placed into ports B-D respectively as per mitostress protocol. 4–8 technical replicates per condition were run. Parameters including response to injection, maximal respiration, and others were calculated as per manufacturer’s instructions.

### Pa growth assay

Two ml overnight LB cultures grown at 37 °C with shaking of indicated Pa strains were normalized to an OD600 of 0.01 in indicated media, then CFTR modulators or equivalent volume of DMSO was added. 100 µL of each culture was placed in a 96-well plate and OD600 was measured each hour by an incubating plate reader at 37 °C.

### Pa motility assay

PA14 wild type and its flgK mutant derivative^32^ were cultured overnight then normalized to OD600 of 0.100. Aliquots of each strain were treated with DMSO or elexacaftor, tezacaftor, and ivacaftor (ETI) then immediately placed onto glass slides, 0.01% Tween-20 was added to reduce surface binding. Images were acquired over 1 min from 63 × oil immersion objective on a Zeiss microscope using an iPhone X. Slow motion images were transferred to ImageJ, and 10-image series were created with 20 slow motion frames between each series image. Manual particle tracking was performed on 18–20 individual bacteria in each series. Each data point represents the total distance (arbitrary units) traveled by each bacterium over the series with lines at the mean.

### Cytokine multiplex assay

MDM were generated at 5 × 10^5^/well in 24 well plates, then treated with CFTR modulators for 48 h as above. Overnight cultures of DH1137 were prepared as in phagocytosis assays on the morning of the experiment. Media was removed from MDM and replaced with antibiotic-free media with fresh CFTR modulators, then DH1137 was added at an MOI of 10 for 2 h. Supernatants were removed from wells, centrifuged for 10 min at max speed in a tabletop microcentrifuge at 4 °C then frozen at − 20 °C until use. Millipore human 48-plex cytokine assay was run by the Dartmouth Immune Monitoring Core Facility.

### Statistical analysis

Analyses were performed in R. ANOVA or mixed effects linear models (where donor was specified as a random effect) were used for analysis as indicated in figure legends. *P* < 0.05 was considered to be statistically significant.

## Results

### CFTR modulators enhance phagocytosis of Pa by both CF and nonCF MDM

**Figure 1:**
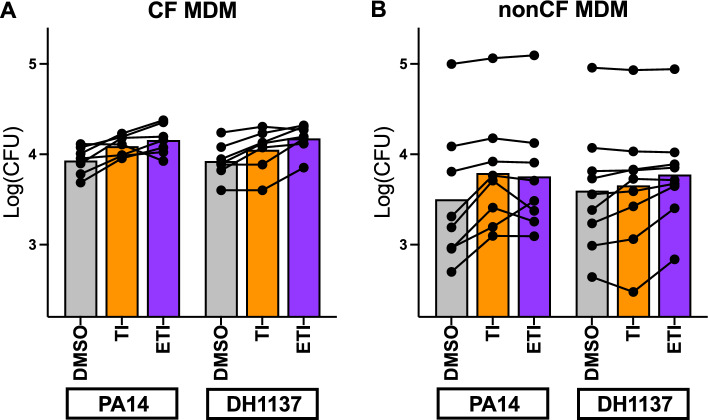
CF and nonCF MDM phagocytosis is augmented by CFTR modulators. (**A**) MDM from CF subjects (n = 7) were treated for 48 h with DMSO or CFTR modulators as indicated. Phagocytosis was measured by gentamicin protection assay. Each line connecting dots represents a single subject, bars represent means of separate experiments. *p* < 0.001 for DMSO versus TI and *p* < 0.0001 for DMSO versus ETI. There were no significant differences between bacterial strains, and no interactions between strain and modulators. (**B**) Identical experiments were carried out with nonCF donors (n = 9). *p* < 0.01 for DMSO versus TI, *p* < 0.0001 for DMSO versus ETI. All statistics by mixed effects linear model.

We first examined the effects of CFTR modulators on one of the prototypical macrophage function, phagocytosis. We utilized two different Pa strains for these assays, one classical laboratory strain (PA14) and one clinical strain isolated from CF lungs (DH1137). We analyzed all results by mixed-effect linear modeling which allowed us to account for subject and Pa strain variability while determining the effects of the modulators globally within the assays. When treated with double CFTR modulator combination TI, CF MDM demonstrated a consistent increase in phagocytosis efficiency (*p* < 0.001 compared with DMSO) (Fig. 1A). Further increases in phagocytosis were seen with triple modulator ETI (*p* < 0.0001).

To investigate the specificity of modulator effects we repeated these studies using nonCF MDM (Fig. 1B). While there appeared to be more variability between subjects, the response to TI remained statistically significant (*p* < 0.01), and similarly to CF MDM, ETI further increased phagocytosis compared with DMSO-treated cells (*p* < 0.0001).

We queried within our model whether there were baseline differences between CF MDM and nonCF MDM with phagocytosis efficiency and did not determine any obvious effects, however given the nature of the assays being carried out on different days, within-assay comparisons are likely more valid than between-assay. We also saw no significant interactions between subject cohort (CF vs. nonCF) and response to CFTR modulators, suggesting that modulator effects may have been independent of CFTR mutation.

There were no significant interactions between the Pa strains in their responses to modulators.

### Mitochondrial function is inhibited by ETI as well as by Pa secreted products

We next explored potential mechanistic factors contributing to phagocytosis efficiency. Macrophage metabolic state impacts their function and vice versa, with pro-inflammatory activated macrophages undergoing a shift toward increased glycolysis and decreased oxidative phosphorylation despite the presence of adequate oxygen^33–36^; this phenomenon is also referred to as aerobic glycolysis or Warburg metabolism. We therefore investigated the metabolic state of CF and nonCF MDM in response to CFTR modulator treatment and secreted factors from PA14 using a Seahorse extracellular flux assay. We chose to use bacteria-free conditioned medium to eliminate metabolic effects of live bacteria confounding the assay.

**Figure 2:**
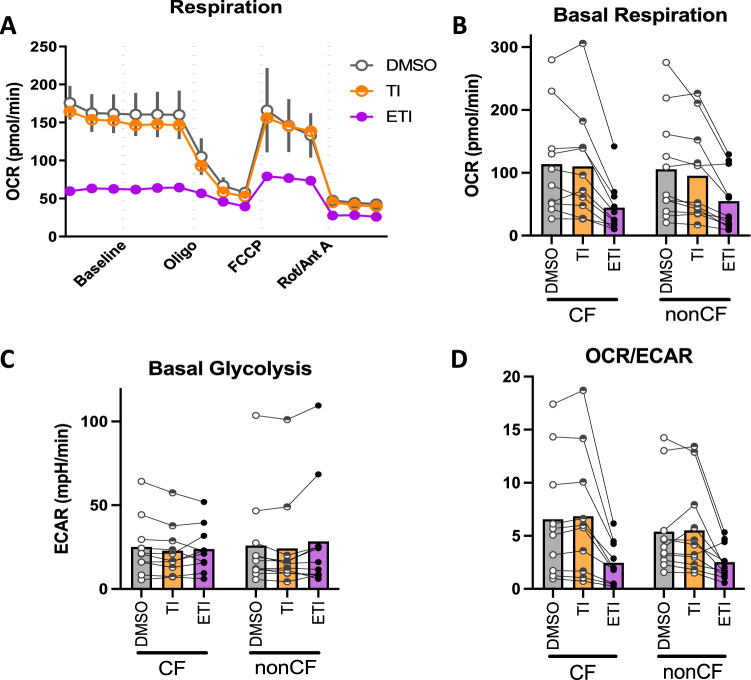
CFTR triple modulators inhibit MDM mitochondrial function. CF and nonCF MDM were treated for 48 h in vitro with CFTR modulators as in previous experiments. A Seahorse mitostress assay was then performed. (**A**) Representative mitostress assay with nonCF MDM pretreated with DMSO, TI, or ETI. (**B**) Basal Respiration of all individual subject MDM tested with and without modulators. Each point with connecting lines represents the mean of technical replicates from an individual subject (CF n = 10, nonCF n = 11). Bars represent means of all the subjects within the group. (**C**, **D**) Basal Glycolysis and OCR/ECAR from the same assays as (**B**).

We found that ETI but not TI inhibited baseline oxygen consumption (a proxy for mitochondrial activity) in both CF and nonCF MDM (*p* = 0.001 by mixed effects linear model, Fig. 2A,B). We did not detect any compensatory increase in basal glycolysis (Fig. 2C). Accordingly, the ratio of mitochondrial activity to glycolysis was consistently diminished in both cohorts in the presence of ETI but not TI (Fig. 2D).

**Figure 3:**
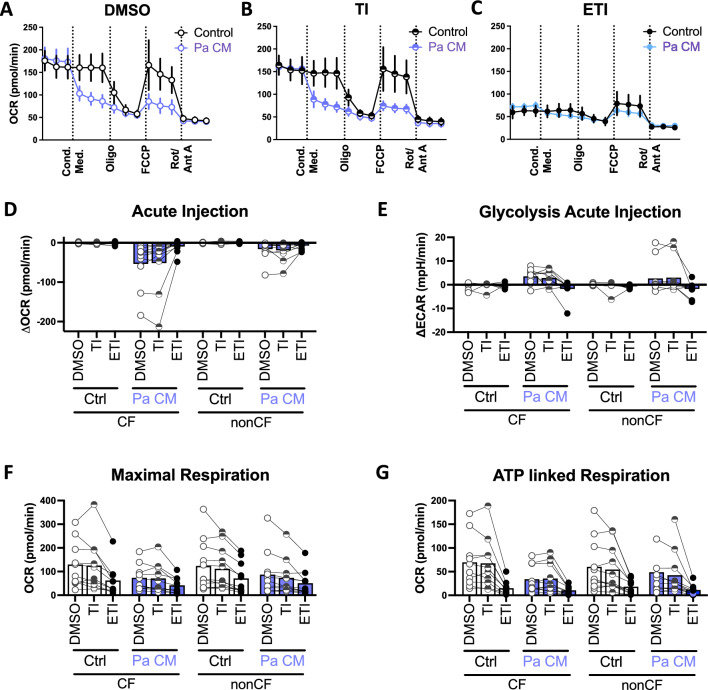
Pa Conditioned Medium inhibits MDM mitochondrial function. Concurrently to the assays in Fig. 2, acute injection of Pa conditioned medium compared with control wells in a Seahorse mitostress assay. (**A–C**) Representative mitostress assay with nonCF MDM pretreated with DMSO, TI, or ETI. (**D–F**) Calculated parameters as indicated based on the mitostress assay. Each point with connecting lines represents the mean of technical replicates from an individual subject (CF n = 10, nonCF n = 11). Bars represent means of all the subjects within the group.

We also queried the response to Pa conditioned medium (CM), in the presence or absence of CFTR modulators. Pa CM caused inhibition of mitochondrial activity, as well as an acute increase in glycolysis (*p* < 0.0001 for both comparisons; Fig. 3A–C, blue lines and Fig. 3D,E), however these effects were abrogated in the presence of ETI where the baseline mitochondrial function was low suggestive of a ceiling effect.

There were corresponding decreases in maximal respiration (*p* < 0.0001 for both Pa CM and ETI relative to controls; Fig. 3F), ATP-linked respiration (*p* < 0.001 for Pa CM, *p* < 0.0001 for ETI, Fig. 3G), and spare respiratory capacity (*p* < 0.0001 for Pa CM, *p* < 0.001 for ETI; Supplemental Fig. 1A).

There were no statistically significant effects on other measured parameters including non-mitochondrial respiration and proton leak (Supplemental Fig. 1B,C, respectively).

Given the effects on mitochondrial function we confirmed that modulators were not inducing cell death (Supplemental Fig. 2); we found no evidence of LDH release in the presence of modulators.

### CFTR modulators do not impact Pa growth or motility

**Figure 4:**
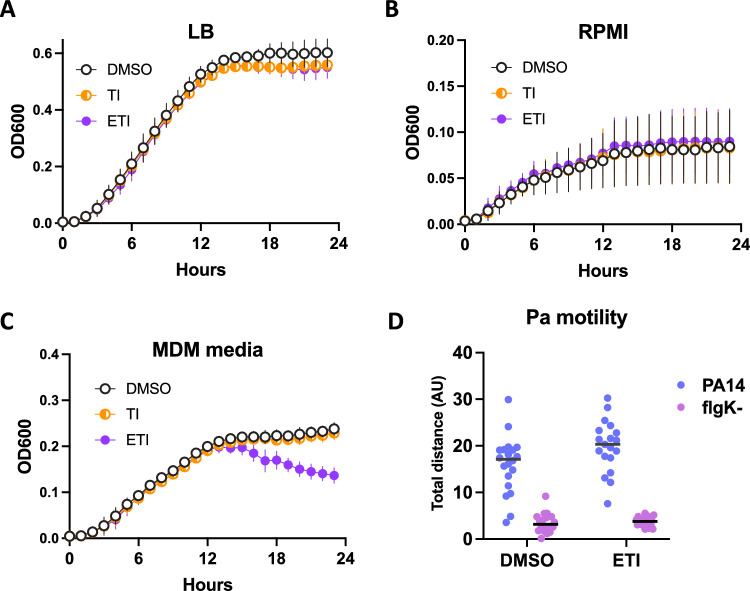
CFTR modulators have no appreciable effect on Pa growth or motility. (**A–C**) Growth of PA14 was measured at 37 °C in a plate reader in various media with or without CFTR modulators added. Points represent means and SEM of three individual experiments, OD600 of media alone without bacteria was subtracted from each data point. (**D**) Motility of the indicated strains was measured on a glass slide by manual particle tracking in the presence or absence of ETI. *FlgK* flagellar mutants were used as a control. Each data point represents the total distance (arbitrary units) traveled by each bacterium over the series with lines at the mean.

It is possible that some of the effects seen on phagocytosis with the gentamicin protection assay could be explained by off-target effects of the CFTR modulators on Pa in vitro. We therefore tested whether CFTR modulators altered Pa growth under the conditions of our phagocytosis assay and found no impact in various media including LB, RPMI or antibiotic-free MDM media (Fig. 4A–C). There was a signal toward decreased bacterial density in the stationary phase > 12 h into the assay, however no differences were seen in the time frame (generally less than 1 h total) of the phagocytosis assays. Pa motility is required for efficient phagocytosis^37^, however CFTR modulators did not appreciably impact motility of PA14, with a *flgK* mutant lacking flagella serving as a non-motile control for comparison (Fig. 4D).

### ETI fails to decrease inflammatory cytokine production in response to Pa infection in CF and nonCF MDM

**Figure 5:**
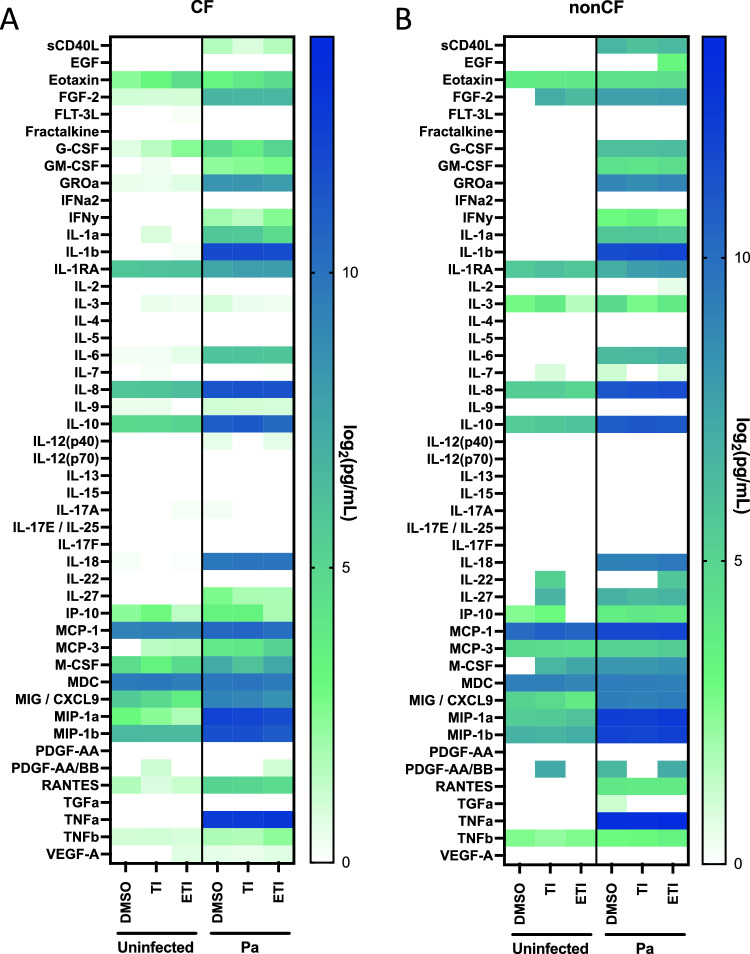
Robust induction of inflammatory cytokines by clinical Pa strain DH1137 is minimally affected by CFTR modulators. (**A**) CF MDM (n = 7) were treated for 48 h in vitro with CFTR modulators, then media was exchanged and a subset were infected with DH1137 at an MOI of 10 for 2 h. Supernatants were spun down and collected. Cytokine multiplex was performed on aggregated samples with 2–4 technical replicates per point. Means for each subject were log-transformed and graphed as indicated. (**B**) nonCF MDM (n = 5) were used in otherwise identical experiments to (**A**).

Early modulators (lumacaftor/ivacaftor) have been reported to mitigate a basal hyperinflammatory state of CF MDM^18^, however the effects of ETI on CF MDM in the presence of Pa have not been studied. We analyzed cytokine production by MDM after infection with DH1137 for 2 h by multiplex assay. In the absence of infection, cytokine secretion was generally low with no discernible effects of TI and ETI. Upon addition of Pa, there was a robust induction of many cytokines in both CF (Fig. 5A) and nonCF MDM (Fig. 5B). Modulators had minimal effect on cytokine production with ETI causing only non-statistically significant increases in inflammatory cytokines such as TNFα, IL-1β, IL-8, IL-10, IL-18, and MCP-1 (Supplemental Fig. 3). There was no indication of a decrease in inflammatory markers with TI or ETI as has been reported with other modulator combinations.

## Discussion

CFTR modulators are known to augment phagocytosis by CF macrophages^18,19,38,39^, and initiation of CFTR modulators in pwCF has been shown to decrease monocyte inflammatory profiles^5,14,15,17,40^. These studies however have not addressed the effects of newer highly effective modulators directly on macrophage function, which is likely distinct from effects on serum monocytes when modulators are initiated in vivo. Indeed, our studies show that while ETI does augment phagocytosis directly in isolated macrophages, these effects appear to be somewhat independent of CFTR mutation, as nonCF MDM also responded (Fig. 1). This is in agreement with a recently published report on the effects of ETI on Burkholderia phagocytosis in isolated MDM by Zhang and colleagues where they also saw effects on nonCF MDM^19^. Interestingly, the authors of that study found variable effects of ETI on killing of Staphylococcus aureus, Pa, and Burkholderia, where nonCF MDM did not respond and CF MDM did. They also found no impact of ETI on inflammatory cytokine production in response to Burkholderia, similar to our results with Pa (Fig. 5) and in contrast to studies showing decreased cytokines with lumacaftor/ivacaftor^18^ and in whole hosts^5,14,15^.

The mechanism underlying the effects of CFTR modulators on nonCF MDM phagocytosis is not entirely clear, and off-target effects were considered. There did not appear to be any major effects of CFTR modulators on Pa growth or motility (Fig. 4) which would explain differences in phagocytic efficiency. It is important to note that these assays were designed to address the question of modulator effects in our in vitro system and do not address the separate question of whether modulators directly impact Pa in the airway, where modulator concentrations are not known but likely to be lower.

Finally, we investigated metabolic effects of CFTR modulators, again discovering unanticipated consequences. Macrophages from F508del/F508del CFTR-mutant mice have been reported to have impaired mitochondrial function relative to WT counterparts^33^. Conversely, Lara-Reyna et al.^41^ found that CF monocytes and MDM have increases in both mitochondrial function and glycolytic activity relative to nonCF when stimulated with IFNγ and LPS, an effect attributed to activation of the unfolded protein response. To our knowledge this is the first study of primary human CF macrophage metabolism and response to CFTR modulators. In contrast to other experimental systems using different cell types^42–44^, in MDM we did not find consistent differences in basal or maximal mitochondrial function between CF and nonCF. We did however find a strong, reproducible inhibition of mitochondrial respiration by triple but not double CFTR modulators (Fig. 2). Presumably this is due to direct activity of the elexacaftor but the mechanism of inhibition is not currently clear. Given that it decreased maximal respiration in the presence of the proton uncoupler FCCP (Fig. 3), it is possible that it directly decreases mitochondrial membrane potential. Notably, effects were similar in both CF and nonCF macrophages. Given that CFTR modulators are of interest in other respiratory diseases such as Chronic Obstructive Pulmonary Disease^45–47^, this may impact understanding of modulator effects outside of CF specifically. Studies are ongoing in our laboratory to further elucidate the mechanism of their mitochondrial inhibition. Intriguingly, Riquelme et al. described colocalization of CFTR signal with mitochondria in airway epithelial cell lines^48^ as well as altered mitochondrial activity profiles in PBMC from CF subjects and in CFTR mutant mice. These effects included increased superoxide and succinate levels. CFTR transfection appeared to improve these phenotypes however the effects of modulators were not tested. Aerobic glycolysis has also been described in CF neutrophils in a manner which resolves after lung transplantation, arguing that the effect is secondary to systemic inflammation rather than CFTR within the neutrophils themselves^49^.

In addition, Pa secreted factors inhibited mitochondrial function (Fig. 3), an effect which has been previously described in different cell types including mouse liver^50^, fibroblasts^51^ epithelial cells^52^, and cancer cell lines^53^.

Aerobic glycolysis in macrophages is classically associated with a pro-inflammatory phenotype, including increased phagocytosis^34,54^, potentially consistent with a model whereby CFTR triple modulators and Pa both activate macrophages and therefore enhance phagocytosis, however this does not explain the effects of TI which had an intermediate phagocytosis phenotype but no effect on metabolism (Figs. 1 and 2). Ultimately there are likely many factors at play resulting in the endpoint of phagocytosis.

A strength of our work is the direct comparison between effects of vehicle, TI, and ETI in parallel in cells from the same subjects. This allows us to tease out specific effects of the modulators more so than studies investigating cellular function before and after initiation of modulators. ETI causes numerous effects including decreased mucus accumulation and subsequent drop in bacterial load which can have indirect downstream effects on monocyte/macrophage function, decreasing overall inflammation within the host and therefore limiting the ability to draw conclusions regarding the direct effects of modulators on immune cells. Our experimental design therefore provides a complementary approach to pre/post ETI human studies allowing interrogation of ETI effects at a finer resolution.

Limitations of this work include that CF volunteers for this study were all taking ETI at baseline, which may have long-lasting effects on monocyte biology including epigenetic modification^13^. In our system however, monocytes are cultured for 7 days during the macrophage differentiation process in vitro prior to re-addition of CFTR modulators, allowing time for washout of the drugs. Others have seen washout of modulator effects in monocytes after 36 h^40^. In addition, the parallel investigation of DMSO versus modulator-treated cells argues for direct effects of the modulators themselves rather than carryover effects from modulator treatment in vivo. Furthermore, the effects on nonCF MDM which have no history of prior modulator exposure provide additional supporting evidence for their short-term effects. Our ex vivo system for investigation of macrophage function is certainly not fully indicative of in vivo conditions where there are many more interactions over an extended period, but it allows for interrogation of those interactions at a finer level which can inform understanding of immune cell and bacterial biology.

In addition, while MDM are more easily obtained and studied than lung macrophages isolated via bronchoalveolar lavage, they carry important differences. Investigations are ongoing in our laboratory to discern whether the effects found here are replicated in primary lung macrophages. Finally, the concentrations of CFTR modulators in the epithelial lining fluid within the alveoli are not known. Concentrations of ivacaftor in sputum have been reported in individual subjects^55,56^ and seem to fall in the range of 10- to 100-fold lower than serum concentrations. We are unaware of any studies which have reported directly the concentration within bronchoalveolar lavage or epithelial lining fluid in human subjects. Lung macrophages exist in a continuum from infiltrating monocytes to differentiated alveolar macrophages^57^, therefore they are likely exposed to serum concentrations of modulators initially, followed by the concentrations within the epithelial lining fluid. We are planning future studies to discern the effects of modulators at various doses on macrophage function, as well as the effects of individual modulators not used in combination.

In summary we have shown that CFTR modulators positively impact primary human macrophage phagocytosis in a surprisingly CFTR mutation agnostic fashion, which was associated with inhibition of mitochondrial function and without substantially altering cytokine secretion. These studies along with future studies on primary lung macrophages probing functional and transcriptional responses to modulators will help elucidate mechanisms of altered phagocytosis in CF, toward the goal of improving pathogen control and decreasing bacterial burden in the CF lung.

## Supplementary Information

Supplementary Information.

## Data Availability

The primary data used to generate the figures in this manuscript are available upon request to DSA.
