# Supplementary material for: Comparative effects of CFTR modulators on phagocytic, metabolic and inflammatory profiles of CF and nonCF macrophages
Source: Sci Rep. 2023 Jul 25;13:11995. doi: 10.1038/s41598-023-38300-9 (PMC10368712; doi:10.1038/s41598-023-38300-9)
Supplement: Supplementary file 1 — Supplementary Information. [file 41598_2023_38300_MOESM1_ESM.docx]

Supplemental Data

Methods

**LDH release assay**

nonCF MDM were generated as in other assays, then treated with CFTR modulators for 48 hours. Medium was then harvested and an LDH assay was run per manufacturer’s instructions (Thermo Scientific 88954). Cells treated with lysis buffer were used as a control to represent 100% cell lysis. Signal from media alone without cells was subtracted from each point.

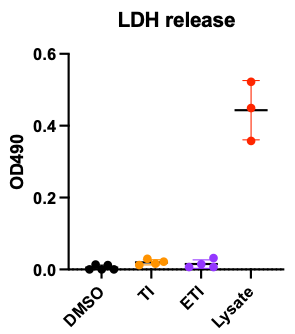


**Supplemental Figure 1. CFTR modulators have no effect on Spare Respiratory Capacity, Proton Leak, or Non-Mitochondrial Respiration in CF or nonCF MDM.** Additional calculated Seahorse Mitostress assay parameters from **Figure 3** are shown. Data are from the same experiments as in **Figure 3.**

**Supplemental Figure 2. CFTR modulators do not cause cell death in MDM.** nonCF MDM were treated for 48 hours with CFTR modulators at previously used concentrations. LDH release assay was then performed, with lysis buffer added to a subset of wells as a positive control. 3-5 technical replicates per condition were run as indicated. Results are representative of three individual experiments.

**Supplemental Figure 3. Selected cytokine data from multiplex experiment in Figure 5.** Data from CF (n=7) or nonCF (n=5) individual subjects are shown with lines connecting dots from a given individual. Points represent means of 2-4 technical replicates per subject. There were no statistically significant differences for TI vs DMSO or ETI vs DMSO for any of the cytokines tested, either with or without Pa infection.

C

D

E

F

A

B
